# Circulating small extracellular vesicles microRNAs plus CA-125 for treatment stratification in advanced ovarian cancer

**DOI:** 10.1186/s12967-023-04774-4

**Published:** 2023-12-22

**Authors:** Xiaofang Zhou, Mu Liu, Lijuan Sun, Yumei Cao, Shanmei Tan, Guangxia Luo, Tingting Liu, Ying Yao, Wangli Xiao, Ziqing Wan, Jie Tang

**Affiliations:** 1 Department of Gynecologic Oncology, Hunan Cancer Hospital, The Affiliated Cancer Hospital of Xiangya School of Medicine, Central South University, Changsha, 410013 People’s Republic of China; 2 Department of Gynecology and Obstetrics, The Central Hospital of Shaoyang, Shaoyang, 422000 People’s Republic of China; 3 Department of Gynecology and Obstetrics, The First People’s Hospital of Huaihua, The Affiliated Huaihua Hospital of University of South China, Huaihua, 418000 People’s Republic of China; 4 Department of Gynecology and Obstetrics, The First People’s Hospital of Changde, Changde, 415000 People’s Republic of China; 5 Department of Gynecology and Obstetrics, The First People’s Hospital of Yueyang, Yueyang, 414000 People’s Republic of China; 6 Department of Gynecologic Oncology, Hunan Gynecologic Cancer Research Center, Hunan Cancer Hospital, The Affiliated Cancer Hospital of Xiangya School of Medicine, Central South University, Address: 283 Tongzipo Road, Yuelu District, Changsha, 410013 People’s Republic of China; 7 Department of Oncology, Xiangya Cancer Center, Xiangya Hospital, Central South University, Changsha, 410008 People’s Republic of China

**Keywords:** Ovarian cancer, Residual disease, Small extracellular vesicles, microRNA, Prediction model

## Abstract

**Background:**

No residual disease (R0 resection) after debulking surgery is the most critical independent prognostic factor for advanced ovarian cancer (AOC). There is an unmet clinical need for selecting primary or interval debulking surgery in AOC patients using existing prediction models.

**Methods:**

RNA sequencing of circulating small extracellular vesicles (sEVs) was used to discover the differential expression microRNAs (DEMs) profile between any residual disease (R0, n = 17) and no residual disease (non-R0, n = 20) in AOC patients. We further analyzed plasma samples of AOC patients collected before surgery or neoadjuvant chemotherapy via TaqMan qRT-PCR. The combined risk model of residual disease was developed by logistic regression analysis based on the discovery-validation sets.

**Results:**

Using a comprehensive plasma small extracellular vesicles (sEVs) microRNAs (miRNAs) profile in AOC, we identified and optimized a risk prediction model consisting of plasma sEVs-derived 4-miRNA and CA-125 with better performance in predicting R0 resection. Based on 360 clinical human samples, this model was constructed using least absolute shrinkage and selection operator (LASSO) and logistic regression analysis, and it has favorable calibration and discrimination ability (AUC:0.903; sensitivity:0.897; specificity:0.910; PPV:0.926; NPV:0.871). The quantitative evaluation of Net Reclassification Improvement (NRI) and Integrated Discrimination Improvement (IDI) suggested that the additional predictive power of the combined model was significantly improved contrasted with CA-125 or 4-miRNA alone (NRI = 0.471, IDI = 0.538, p < 0.001; NRI = 0.122, IDI = 0.185, p < 0.01).

**Conclusion:**

Overall, we established a reliable, non-invasive, and objective detection method composed of circulating tumor-derived sEVs 4-miRNA plus CA-125 to preoperatively anticipate the high-risk AOC patients of residual disease to optimize clinical therapy.

**Supplementary Information:**

The online version contains supplementary material available at 10.1186/s12967-023-04774-4.

## Introduction

Approximately 75% of ovarian cancer cases are not detected until stage III-IV, leading to a 5 year survival rate of less than 30% [1]. Although the best timing for surgery has been controversial, no residual disease (R0 resection) following primary debulking surgery (PDS) is recognized as the most potent determinant of clinical prognosis in advanced ovarian cancer (AOC, high-grade serous ovarian cancer (HGSOC) with FIGO stage III or IV) [2, 3]. Two famous randomized clinical trials have confirmed that neoadjuvant chemotherapy followed by interval debulking surgery (NACT-IDS) has similar progression-free survival and overall survival with fewer surgical complications compared to PDS for AOC patients who cannot achieve R0 resection [4]. In this context, the preoperative identification of patients with unresectable tumors is of utmost importance to optimize the therapeutic choice between PDS and NACT. For commonly used clinical models, CA-125 has no accurate predictive threshold because the preoperative level cannot fully reflect the tumor progression [5, 6], and the radiological evaluation is subjective to some extent. Moreover, using the classic Fagotti laparoscopy scoring [7, 8] as a minimally invasive examination, with a 40.5% unsuccessful prediction operations rate, can lead to a delay in starting chemotherapy and may also facilitate tumor implantation metastasis in puncture sites [9].

At present, biomarkers of liquid biopsies to predict which AOC patients will potentially profit from PDS or NACT therapy are missing. Small extracellular vesicles (sEVs) are membrane vesicles with a diameter of less than 200 nm, which contain specific cargoes that represent selected portions of the source cell's contents, strongly biasing toward microRNAs (miRNAs) [10]. Mounting evidence suggests that sEVs-derived miRNAs could be used for cancer detection, and prognosis, and to guide therapy [11, 12]. Circulating concentrations of sEVs miRNAs vary in response to OC stages [13, 14], and their stability in stored samples makes them plausible candidates as biomarkers.

In this study, we assessed the diagnostic performance of circulating sEV-miRNAs plus CA-125 to distinguish high-risk AOC patients of residual disease for the purpose of treatment stratification into PDS or NACT-IDS.

## Materials and methods

### Clinical samples and ethics approval

A total of 221 AOC patients (HGSOC with FIGO stage III or IV) who were treated with PDS or NACT-IDS from January 2018 to June 2022 were recruited in this study (R0, n = 99; non-R0, n = 122). In addition, we obtained plasma samples from benign pelvic diseases (n = 21), early-stage ovarian cancer with FIGO stage I or II (n = 20), and advanced colorectal cancer (n = 22) patients. We also collected primary tumor tissues (n = 58) and other site tissues (n = 18) from these AOC patients after PDS. None of the patients involved had infectious diseases. AOC samples with the following characteristics were removed: (1) treatment with surgical operation or chemotherapy before plasma collection; (2) hemolysis. Each participant signed a written informed consent form. The study obtained ethical approval (No. KYJJ-2019-043) from the Hunan Cancer Hospital Institutional Review Board. The CA-125 level of each AOC patient was measured by chemiluminescence assay (Beckman, DXI800, CA, USA) before treatment at four clinical centers. Blood samples (8 mL) from every individual in a fasting state were collected with an EDTA-K2 anticoagulant tube in the early morning, stored at 4 °C, and then processed within 30 min. The samples were extracted via centrifugation at 2000 ×*g* for 10 min, and 13,000 ×*g* for 10 min at 4 °C to exclude effects from platelet-derived vesicles as described in refs. [15, 16]. Then the isolated plasma was aliquoted into 2 mL tubes for storage at − 80 °C. Samples from other centers were also processed as described above and then transported via dry ice.

### Isolation and purification of sEVs from plasma

sEVs were purified from 2 mL of plasma from AOC patients by differential ultracentrifugation [16]. After the plasma sample was thawed on ice, centrifuged at 3000 ×*g* for 15 min. The supernatant was carefully pipetted into a new tube and diluted with PBS to 23 mL, and centrifuged at 13,000 ×*g* for 30 min (Beckman Coulter, Brea, CA, USA). Through a 0.22 μm filter, the supernatant was ultracentrifuged at 100,000 ×*g* for 2 h at 4 °C to collect a pellet of sEVs. The pellet was dissolved with PBS, transferred to a new ultracentrifuge tube and centrifuged again at 100,000 ×*g* 4 °C for 2 h to eliminate any contaminants from the protein aggregates. Finally, the enriched pellet of sEVs was resuspended twice with 100 µL PBS and then collected into a new tube. The isolation method of plasma sEVs was performed strictly according to the MISEV2018 guidelines [17].

### Isolation and purification of sEVs from tissue

Primary tumor tissue, matched adjacent tissue, metastatic tumor tissue, and distant normal tissue were collected from AOC patients undergoing PDS. After collecting the living tissue, residual blood was washed with PBS, cut into 500 mg per block, and then placed in a frozen tube. The tissue was quickly transferred into liquid nitrogen flash-frozen for 1 h, and then stored at − 80 °C. Tissue sEVs were separated based on the protocol previously established by Vella et al. [18]. with some modifications [19]. Tissue dissociation was performed using the Miltenyi Human Tumor Dissociation Kit (Miltenyi Biotec, No. 130-095-929, Germany). Before extraction, it was resuspended by enzymes H, R, and A, according to the instructions, and the dissociated mixture containing 2.2 mL RPMI, 100 μL enzyme H, 50 μL enzyme R, and 12.5 μL enzyme A was prepared. A small piece of tissue (~ 200 mg) was weighed, and ultrathin sections were taken using a frozen slicer (to minimize foreign contamination caused by tissue cell rupturing, thereby enlarging the surface area). Then, the dissociation mixture prepared above was added and incubated at 37 °C for 15 min to enzymatic dissociation and permeabilize the tissue. Halt protease and phosphatase inhibitor single-use cocktail, EDTA-free (100X) (Thermo Scientific, No. 78443, USA) was added into dissociation solution and gently filtered twice through a 70 μm filter to remove residual tissue.

The mixed suspension was centrifuged at 300 ×*g* for 10 min at 4 °C. The supernatant was transferred to a new centrifuge tube and centrifuged at 2000 ×*g* for 10 min at 4 °C. The cell-free supernatant was centrifuged at 10,000 ×*g* for 20 min at 4 °C and then gently passed through a 0.22 μm filter to remove remaining cell debris. The supernatant underwent additional ultracentrifugation at 150,000 ×*g* for 2 h at 4 °C. The precipitates were collected and resuspended in 1 mL PBS, and further purified by an Exosupur column (Echobiotech, China). Finally, the sEVs-containing fraction was condensed to 200 μL via a 100 kDa Amicon Ultra ultrafiltration centrifuge tube (Merck, Germany).

### Separating sEVs by immunoaffinity magnetic bead sorting system (MACS)

Plasma and tissue sEVs were isolated via the above methods. Then, 20 μL of magnetic microbeads with antibodies (EpCAM, FAP, CD31, CD235a, CD45; Miltenyi Biotec) was added to 100 μL of sEVs suspension. After incubation for 60 min at 4 °C, the magnetic immune mixture was resuspended with 1 mL of PBS. The LD column (Miltenyi Biotec) was placed in a magnetic rack, and the column was washed three times with 2 mL PBS. The magnetic immune mixture was added to the LD column and then washed three times with 1 mL PBS to clean unlabeled sEVs. The LD column was removed and placed in a 15 mL collection tube. PBS (3 mL) was added to the column, and the magnetic mixture containing labeled sEVs was pushed into the tube by a plunger. The mixture was centrifuged at 100,000 ×*g* for 70 min, and the precipitates were resuspended in 100 μL of PBS. Then, 100 μL of elution buffer (0.1 M glycine pH 2.8) was used to dilute the labeled sEV suspension, which was vortexed for 30 s, and combined with 10 μL of renaturation buffer (1 M Tris–HCl, pH 7.4). The mixture was centrifuged at 10,000 ×*g* for 30 min to separate the sEVs from magnetic beads. The supernatant containing specific sEVs was stored at − 80 °C for RNA extraction.

### Transmission electron microscopy (TEM)

sEVs fixed in 1% paraformaldehyde for 10 min and washed with deionized water. The sEV suspension (10 μL) was placed over a Formvar-carbon-coated 300-mesh copper net and incubated for 10 min at room temperature. After washing with deionized water, the sEVs were negatively stained with 2% uranyl oxalate solution for 1 min at room temperature. The grids were dried for 5 min under incandescent light. Images of sEVs were acquired with an FEI Tecnai G2 Spirit Transmission Electron Microscope (TEM) (FEI; Houston, TX, USA).

### Nanoparticle tracking analysis (NTA)

Based on the characteristics of the Brownian motion and light scattering, the hydrodynamic diameter and concentration of sEVs were measured by NTA (ZetaView PWX 110, Particle Metrix, Germany). SEVs were diluted to 1 mL with PBS and injected into the cuvette. The hydrodynamic diameter and concentration of particles were calculated from the diffusion coefficient by the Stokes–Einstein equation. Five videos of approximately 10 s duration each were recorded for every sample, and analysis of the data regarding particle movement was by the nanoparticle tracking analysis (NTA) software.

### Western blotting

Protein quantification of sEVs samples was conducted using the BCA Protein Assay Kit (Thermo Fisher Scientific, No. 23,225, USA). The primary antibodies used were CD9 (Abcam, ab92726), CD63 (Abcam, ab216130), CD81 (Abcam, ab109201), TSG101 (Abcam, ab125011), HSP70 (Abcam, ab2787), calnexin (Abcam, ab22595), EpCAM (Abcam, ab32392), FAP (Abcam, ab207178), CD31 (Abcam, ab9498), CD45 (Abcam, ab40763), and β-actin (Proteintech, 66,009–-1-lg). For secondary antibodies, goat anti-rabbit (A0208, Beyotime) and goat anti-mouse (A0216, Beyotime) IgG horseradish peroxidase were used.

### High-throughput sequencing and differential expression analysis

Total RNA extracted from sEVs of 2 mL plasma was used for miRNA library preparation (Ribobio, China). The clean reads (17–45 nt) were contrasted with human genome databases (Silva, GtRNAdb, Rfam, and Repbase) using the Bowtie software [20]. The clean reads were further compared with mature miRNAs in the miRDeep2 and miRBase databases. Next, the miRNA expression levels were normalized by the RPM. The significant differential expression analysis of miRNAs was conducted using the edgeR and limma packages.

### RNase treatment of sEVs and RNA extraction

The plasma from the same patient was divided into 3 equal parts. One of which extracted sEV fractions was treated with RNase A (10 μg/ML, Tiangen, No. RT405, China), which is used to eliminate the free RNA carried by non-vesicles, for 15 min at 37 °C. The remaining two plasma samples were not digested with RNase, one of which was directly used to extract plasma RNA, and the other was used to extract RNA from sEVs.

According to the standard protocol, total RNA from plasma or sEVs was extracted and purified by the miRNeasy Serum/Plasma Advanced Kit (Qiagen, No. 217,204, USA). Total RNA from tissue was extracted via a miRNeasy Mini Kit (Qiagen, No. 217004, USA). RNA degradation and contamination were detected by gel electrophoresis. The integrity of RNA was assessed on an Agilent 2200 TapeStation (Agilent Technologies, CA, USA). Additionally, RNA samples were quantified by Qubit^®^2.0 (Life Technologies, USA).

### Quantitative real-time PCR (qRT-PCR)

*C. elegans* cel-miR-39 was used as an external calibration, and U6 was used as an internal reference for tissue and tissue sEV samples. miRNA quantification was performed by a Light Cycler 480 (Roche, Germany) using specific miRNA TaqMan gene expression probes (Synbio Technologies, China) mixed with cDNA templates (Takara, RR037A, Japan). The relative expression levels of candidate miRNAs were normalized to the control group via the Eq. 2-ΔΔCt [21]. The primer and probe sequences of the miRNAs were listed in Additional file 1: Table S3.

### Pathway enrichment analysis

Target genes of miRNAs were screened via TargetScan and miRWalk. The Tothill dataset (GSE61568) [22] was used to establish the miRNA-gene regulatory network and perform pathway analysis. Tumor cases with low malignant potential, non-serous histology, or low grade from the Tothill dataset were excluded. Samples that received NACT or did not provide residual disease status were excluded. Accordingly, miRNA target genes were matched using only data from primary tumors of patients with HGSOC undergoing surgery in the Tothill dataset. The DAVID website (https://david.ncifcrf.gov.) was used to conduct functional clustering and enrichment pathway analysis. We classified the GO and KEGG pathways using the two-sided Fisher's exact test and calculated FDR to correct P values. A corrected P value < 0.05 was considered to be significantly enriched in the GO/KEGG pathway. GSEA was performed by GSEA software (http://software.broadinstitute.org/gsea/).

### Statistical analysis and prediction model building

LASSO analysis was performed to select the most relevant predictors via the "glmnet" (strictly set alpha = 1) package. Based on the R0 group as a control, we calculated the relative expression abundance of selected miRNAs in the non-R0 and differential diagnostic groups (BPD, ESOC, ACC). Log2 transformation of 4-miRNA qRT-PCR expression values of all samples was completed using the log (x + 1, 2) formula [11, 23]. Serum CA-125 expression values were converted via log10. We preliminarily fitted the model using logistic regression, as described in refs. [11, 24]. Based on this multivariate logistic regression model, we calculated the risk probability of residual disease for each subject through leave-one-out cross-validation. Moreover, the index score of differential diagnostic patients was also calculated by this risk model. We assessed the optimal cutoff value of the above prediction probability (4-miRNA combined CA-125) by receiver operating characteristic (ROC) curves and calculated sensitivity, specificity, AUC, PPV, and NPV in the discovery set [23]. While using the same cutoff value of risk prediction, the corresponding sensitivity, specificity, AUC, PPV, and NPV were calculated by the ROC curve in the validation set. The NRI and IDI were calculated by R software to quantify the improvement of diagnostic separation [25]. Furthermore, DCA was conducted by the “rmda” package in R. Gpower software was used to calculate the post hoc power of the prediction model. Multivariate and univariate logistic regression were employed to analyze the influence of various clinicopathological variables on residual disease status, including age, stage, lymph node metastasis, TP53 mutation, serum CA-125 levels, and 4-miRNA panel.

For all cohorts, MedCalc statistical software, version 19.1.0 (Medcalc Software, Ostend, Belgium) was used for the calculation and visualization of ROC curve relative indicators [24]. Statistical analysis was carried out using SPSS, GraphPad Prism 8, and R software (version 3.6.3). Comparisons between groups were performed using an unpaired t test or Mann–Whitney U test. The limit of significance was defined as a P value < 0.05. The results were visualized via the R packages VennDiagram, pheatmap, GOplot, and ggplot2.

### Data availability statement

RNA sequence data of all samples were publicly stored in the Gene Expression Omnibus of NCBI, GSE223126 (https://www.ncbi.nlm.nih.gov/geo/query/acc.cgi?acc=GSE223126). The accession numbers of previously published datasets involved in our study were GSE113486 [26], GSE94533 [27], and GSE61568 [22].

## Results

### Assay design and clinical characteristics

**Fig. 1:**
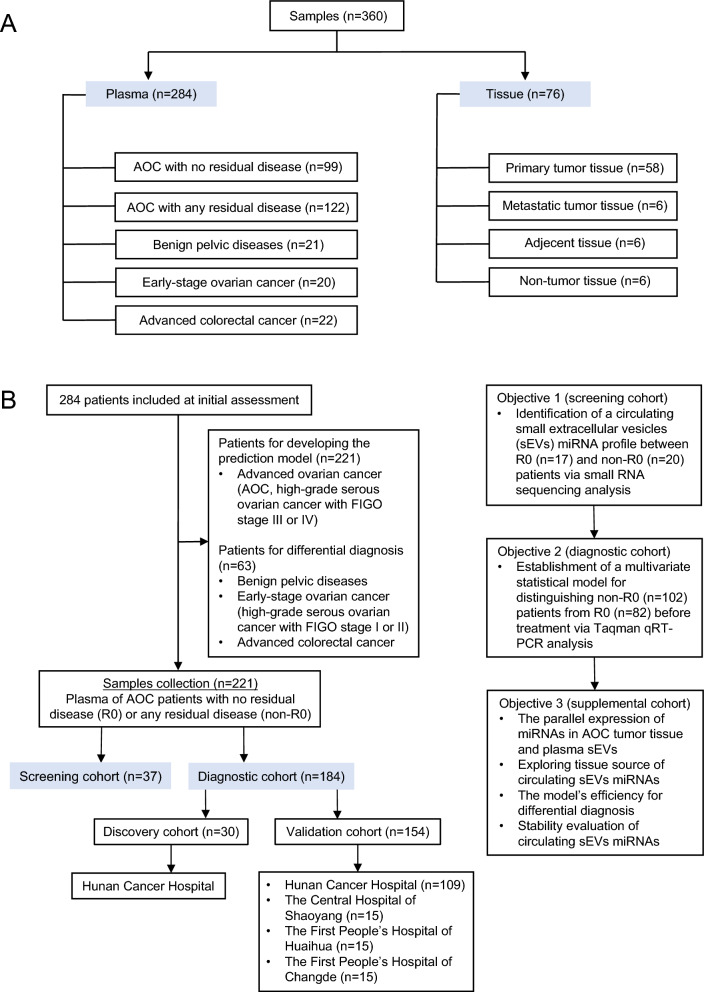
Assay design and clinical characteristics A: **A** total of 360 plasma and tissue samples were tested in our study. **B**: Flowchart of study design was prepared to establish a diagnostic model for predicting residual disease risk in AOC patients. *AOC* advanced ovarian cancer

A total of 284 pretreatment plasma samples and 76 surgical tissue samples were collected in this study (Fig. 1A). We sought to find a reliable panel for residual disease risk detection in AOC patients. Our design of the three-phase study is shown in Fig. 1B, Additional file 1 Figure S1. Concretely, a total of 284 patients from 4 clinical centers were included in the initial analysis, including 99 AOC patients without any residual disease (R0), 122 AOC patients with any residual disease (non-R0), 20 early-stage OC patients, 21 benign pelvic disease patients, and 22 advanced colorectal cancer patients. Using these samples, we constructed three cohorts: a screening cohort (n = 37), a discovery cohort (n = 30), and a validation cohort (n = 154).

### Plasma sEV-derived miRNA profile in AOC patients

**Fig. 2:**
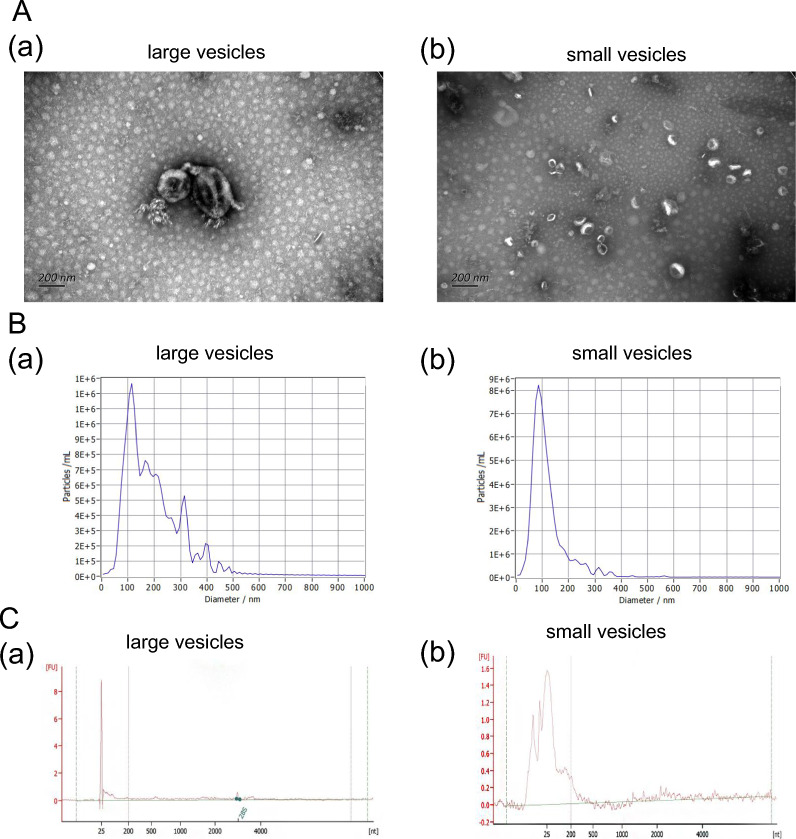
Characterization of large and small EVs isolated from AOC tissues. **A**: 10 micro-liters of large **a** and small vesicles **b** from AOC tissues were loaded onto grids, negative stained, and evaluated with transmission electron microscopy (TEM). Scale bars, 200 nm. **B**: Size distribution of large **a** and small vesicles **b** were obtained using nanoparticle tracking analysis (NTA; ZetaView^®^). Size distribution is presented as graphs with the concentration of the structures on the y-axis and the diameter of the structures in nanometres on the x-axis. **C:** RNA of large **a** and small vesicles b were isolated directly from the EV pellets and was analysed with a Bioanalyzer^®^ (Agilent 2100). *AOC* advanced ovarian cancer

To focus on the sEVs released from AOC patients, we first determined the presence of vesicles within the plasma of AOC patients using electron microscopy. Transmission electron microscopy (TEM) images showed that large and small vesicles isolated from AOC patients' plasma had completely different sizes (Fig. 2A). There were also obvious differences in the diameter ranges of large and small vesicles via nanoparticle tracking analysis (NTA) (Fig. 2B). Furthermore, we found that the large vesicles harbored visible peaks for the 28S ribosomal RNA and a narrower peak for small-RNAs, while small vesicles had a broader small-RNA peak as determined by high-resolution electrophoresis (Agilent 2100 Bioanalyzer) (Fig. 2C). The typical sEV marker proteins CD9, CD63, CD81, TSG101, and HSP70 were also detected in our isolated vesicles, whereas the negative marker protein, calnexin, was absent (Additional file 1: Figure S2). These data proved that the extracted vesicles were extracellular and that miRNAs were mostly derived from small extracellular vesicles (sEVs).

**Fig. 3:**
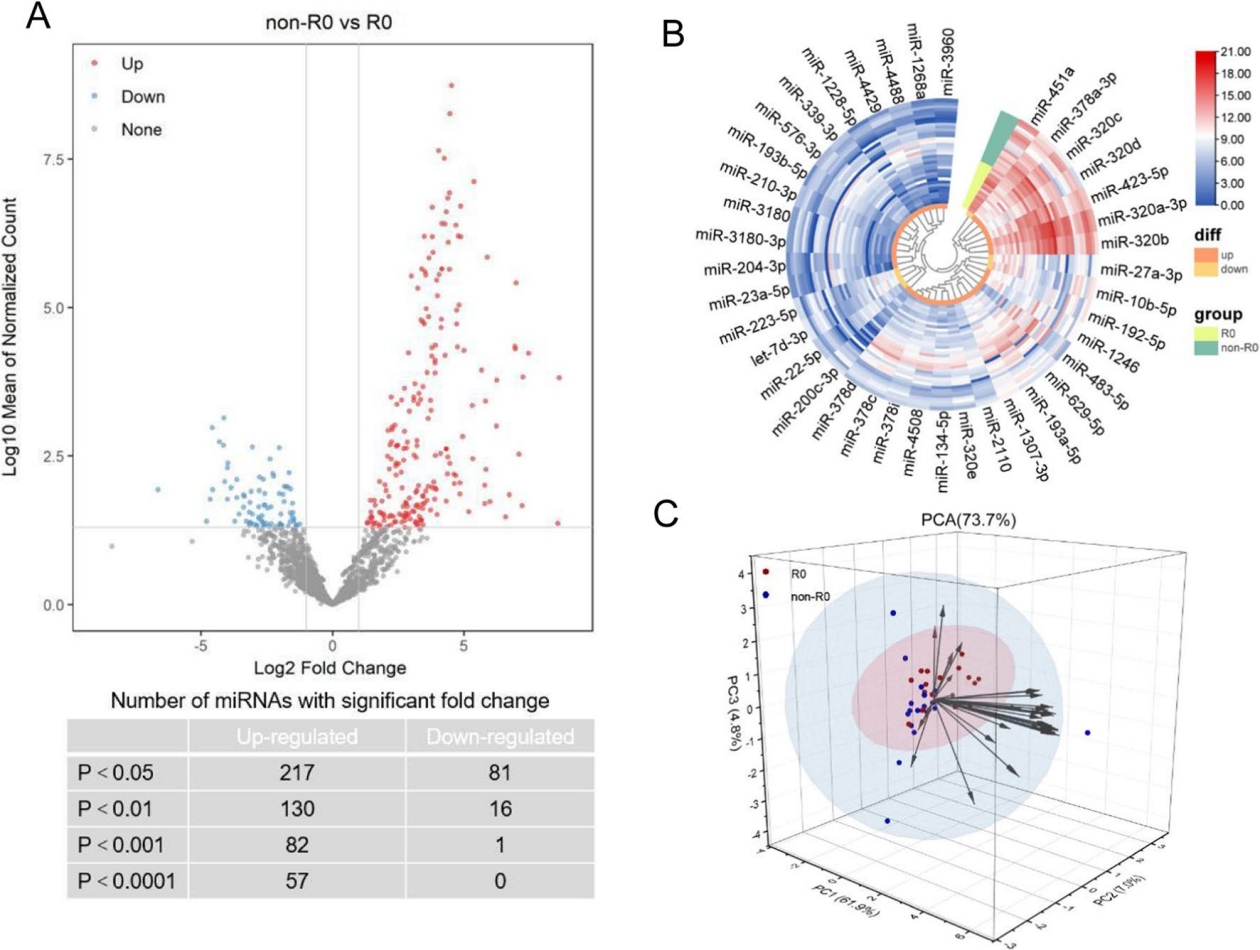
Plasma sEVs derived miRNA profile in AOC patients **A**: Volcano plot showed all DEMs between non-R0 (n = 20) and R0 (n = 17) groups in small RNA sequencing. The red and blue represented the up-regulated and down-regulated DEMs, respectively. (|log2(FC)|> 1, P < 0.05); The table summarized the numbers of DEMs defined by different P values. **B**: A heatmap of 51 DEMs expressions in miRNA-seq data. **C**: Principal component analysis of 51 DEMs expressions between non-R0 and R0 groups. *miRNA* microRNA, *sEVs* small extracellular vesicles, *AOC* advanced ovarian cancer, *DEMs* differentially expressed miRNAs, *R0* advanced ovarian cancer with no residual disease, *non-R0* advanced ovarian cancer with any residual disease

To identify a global expression profile of circulating sEVs-derived miRNAs between non-R0 and R0 patients, we tested 37 AOC patient samples in the screening set (R0, n = 17; non-R0, n = 20) using small RNA sequencing. In all samples, we detected 1832 known miRNAs, and 151 differentially expressed miRNAs (DEMs) between the two groups (|log2(FC)|> 1 and P < 0.05) (Fig. 3A). To avoid the bias in the differential analysis caused by miRNAs with low expression, 51 DEMs (42 upregulated; 9 downregulated) with an average RPM > 50 was chosen from 151 DEMs as candidate miRNAs (Fig. 3B). The log (FC) value and the P value of these 51 selected DEMs are included in Additional file 1: Table S1. Unsupervised cluster heatmap and principal component analysis (PCA) further identified that these 51 DEMs could distinguish non-R0 subjects from R0 subjects (Fig. 3B, C). Moreover, the driver genes of HGSOC (TP53, PTEN, BRAC, RB1, etc.) have been recognized and published in Nature [28, 29]. 15 miRNAs targeting these driver genes were further filtered from 51 DEMs.

### Construction of a prediction model using plasma sEV-derived miRNAs and CA-125 in the discovery set

**Fig. 4:**
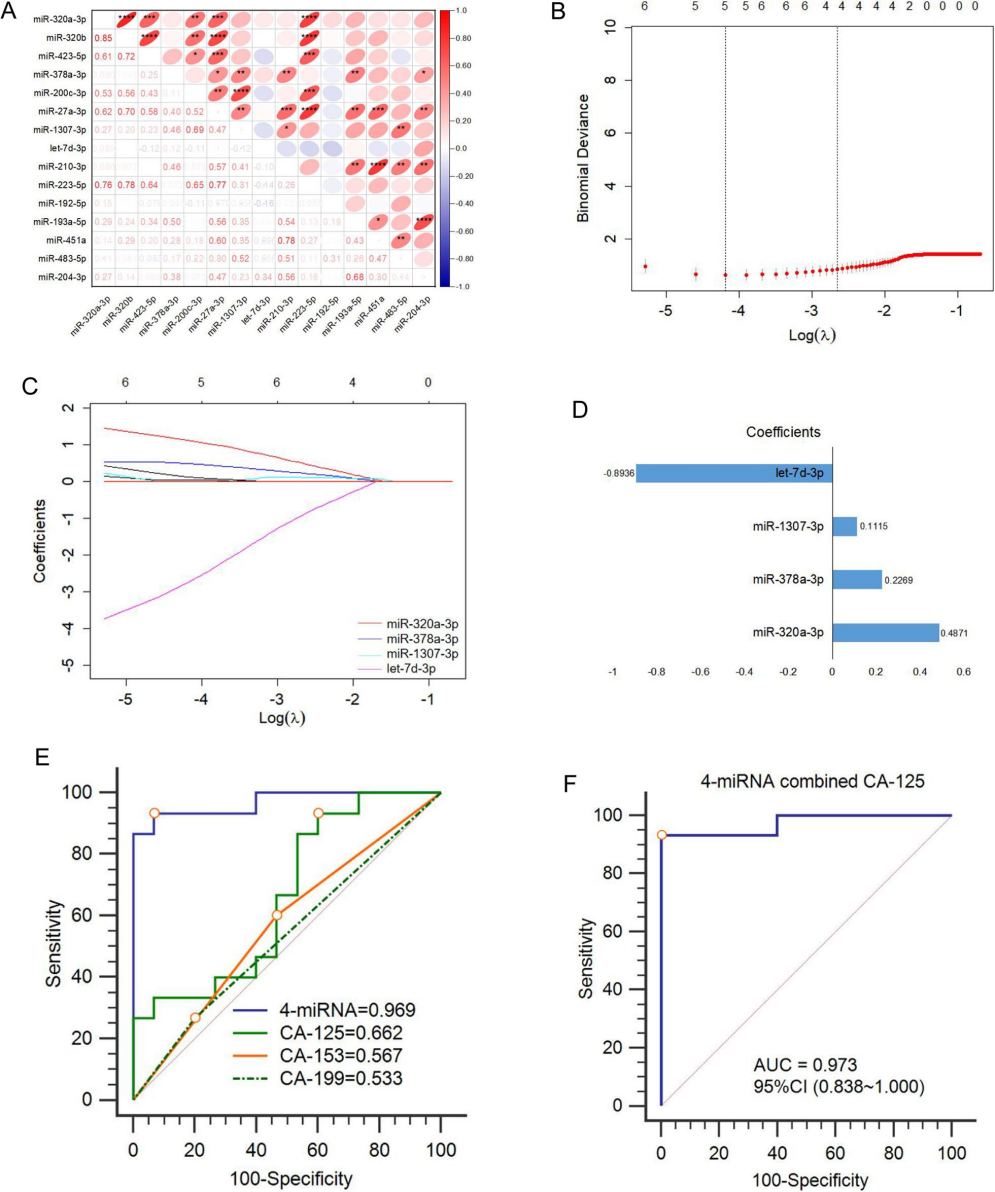
Construction of a prediction model using plasma sEVs derived miRNAs and CA-125 in the discovery set **A**: Pearson correlation analysis of 15 selected miRNAs levels detected by Taqman qRT-PCR in the discovery set (R0, n = 15; non-R0, n = 15). Pearson correlation coefficient and P value were displayed in the bottom-left and the upper-right, respectively. **B**: The log(λ) was plotted versus AUC. Numbers along the upper x-axis indicated the number of predicted factors. The black vertical lines defined the optimal values of λ (λ = 0.07), where the model provided the best fitting to the data; **C:** The LASSO coefficient profile plot of the selected 4 texture features (miR-320a-3p, miR-378a-3p, miR-1307-3p, let-7d-3p). **D**: The LASSO coefficient values of 4 miRNAs. **E–F**: The ROC curves of (**E**) 4-miRNA panel, CA-125, CA-153, CA-199, and **F** 4-miRNA combined with CA-125 for detecting residual disease in the discovery set. Maximum classification accuracy was labeled by the red circle. *miRNA* microRNA, *sEVs* small extracellular vesicles, *R0* advanced ovarian cancer with no residual disease, *non-R0* advanced ovarian cancer with any residual disease, *LASSO* least absolute shrinkage and selection operator, *AUC* area under the receiver operating characteristic curve, ROC receiver operating characteristic curve; *P < 0.05; ** < 0.01; *** < 0.001; **** < 0.0001

In the independent discovery set consisting of 30 AOC patients (R0, n = 15; non-R0, n = 15), we examined the diagnostic robustness of the above 15 selected miRNAs by TaqMan qRT-PCR for further screening of miRNAs with expressed commonality in order to exclude the influence of individual differences. The qRT-PCR data illustrated that 15 miRNAs had a potential co-regulation (r > 0.6) (Fig. 4A). The fold change, P value, and area under the receiver-operator characteristic (ROC) curve (AUC) of 15 miRNAs are shown in Additional file 1: Table S2. Then, we screened 8 miRNAs with significant differences from 15 miRNAs in the discovery set (Additional file 1: Table S2). Since the diagnostic performance of a single miRNA was relatively low (AUC: 0.58 ~ 0.78), we planned to use multiple miRNAs combined with clinical serum biomarkers to develop the prediction model.

To obtain better performance parameters and avoid overfitting the model, the best risk /predictive panel containing 4 miRNAs from the above 8 miRNAs was selected by least absolute shrinkage and selection operator (LASSO) regression (penalized coefficients, λ = 0.07) (Fig. 4B, C). The LASSO coefficient of 4-miRNA is displayed in Fig. 4D. Consistent with clinical application, CA-125 is an initial and important clinical biomarker of AOC [30], as CA-125 (AUC: 0.662) showed relatively higher diagnostic performance than CA-153 (AUC: 0.567) and CA-199 (AUC: 0.533) in distinguishing non-R0 from R0 patients (Fig. 4E). Interestingly, a new combination signature of 4-miRNA and CA-125 (AUC: 0.973, 95% CI 0.838 ~ 1.000) demonstrated a higher detection accuracy than the 4-miRNA panel (AUC: 0.969) or CA-125 alone (AUC: 0.662) (Fig. 4E, F), just as serum CA19-9 could also improve the diagnostic performance of the miRNA biomarker panel for detecting gastric cancer [31] or pancreatic adenocarcinoma [32]. The indicators related to the ROC of the above predictors are shown in Table 2. The risk score model of 4-miRNA and CA-125 was established by logistic regression analysis as follows: Index mC = miR-320a-3p*(0.179) + miR-378a-3p*(0.067) + miR-1307-3p*(0.052) + let-7d-3p*(− 0.198) + CA-125*(0.122) + 0.701 (cutoff = 1.483). If the index mC of AOC patients is higher than the cutoff value, they would be defined as high-risk patients with residual disease.

### Combining 4-miRNA with CA-125 for R0 and non-R0 patient categorization in the validation set

**Fig. 5:**
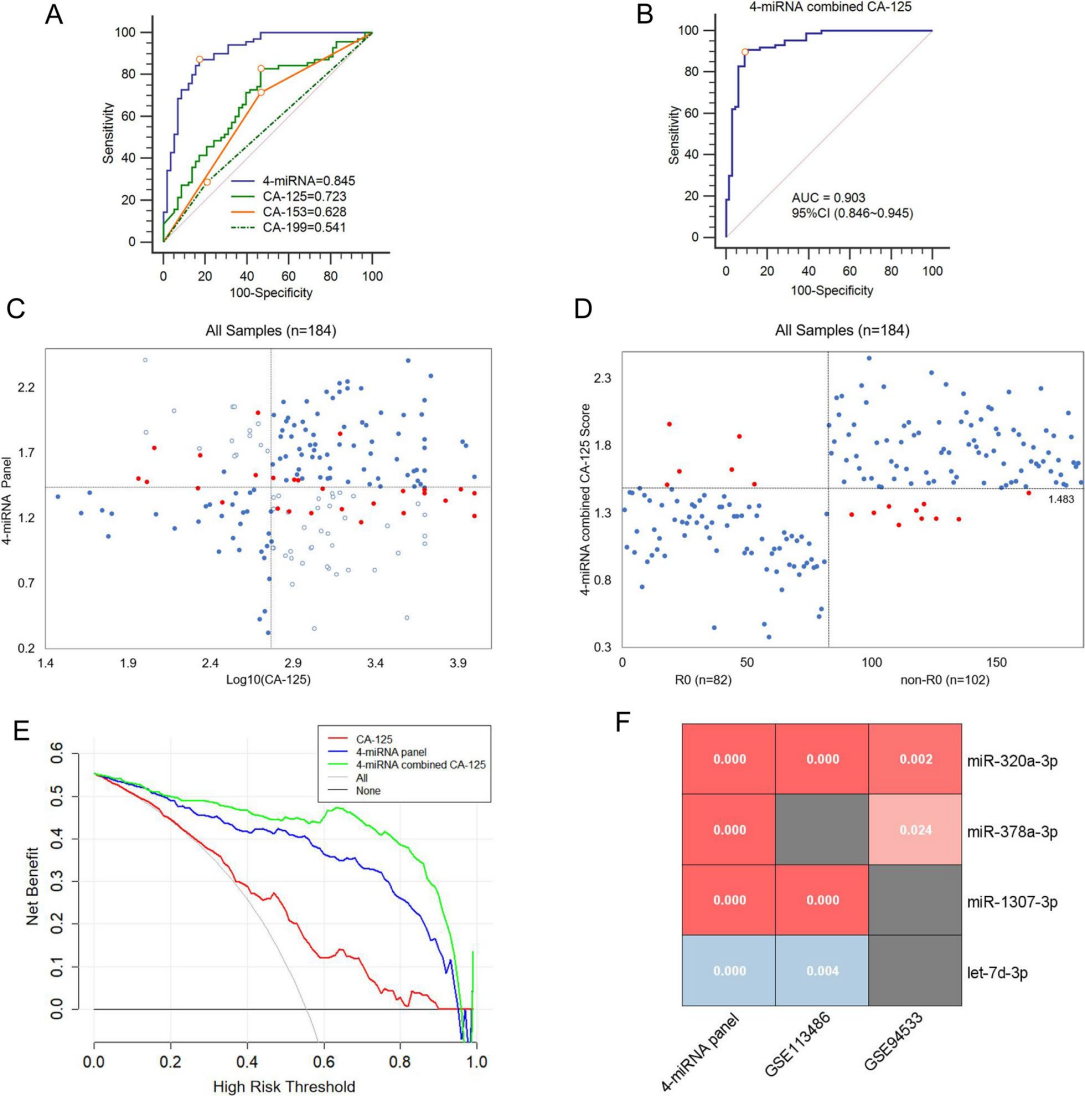
Combining 4-miRNA with CA-125 for R0 and non-R0 patients categorization in the validation set **A**–**B**: The ROC curves of **A** 4-miRNA panel, CA-125, CA-153, CA-199, and **B** 4-miRNA combined with CA-125 for detecting residual disease in the validation set (R0, n = 67; non-R0, n = 87). Maximum classification accuracy was labeled by the red circle. **C:** The 2-dimensional classified plot of the 4-miRNA panel score (y-axis) and serum log10(CA-125) level (x-axis) for all subjects in the discovery and validation sets (n = 184). The horizontal and vertical dashed lines represented the classification threshold of the 4-miRNA panel (1.422) and CA-125 (600 U/ml), respectively. The misclassified cases via 4-miRNA panel or CA-125 were marked with a red point (n = 27) or a blue circle (n = 59), respectively. **D**: The 2-dimensional classified plot of the prediction model combining 4-miRNA with CA-125 for all subjects in the discovery and validation sets (n = 184). The horizontal dashed line was the classification threshold (1.483) of the combined model. The false-positive and false-negative cases were in red (n = 16). **E**: The decision curve analysis (DCA) plot of three models (CA-125, 4-miRNA, 4-miRNA combined with CA-125) (n = 184). **F**: The P value list of differentially expressed 4-miRNA in our panel and public datasets(GSE113486, GSE94533). Red marked up-regulated miRNAs and blue marked down-regulated miRNAs. miRNA. microRNA; *ROC* receiver operating characteristic curve, *R0*, advanced ovarian cancer with no residual disease, *non-R0* advanced ovarian cancer with any residual disease; *P < 0.05; ** < 0.01; *** < 0.001; **** < 0.0001

Next, we evaluated the accuracy of this statistical model in the independent validation set (R0, n = 67; non-R0, n = 87). Compared to 4-miRNA, CA-125, CA-153, or CA-199 alone, the combined model had the highest diagnostic utility (AUC: 0.903, 95% CI 0.846 ~ 0.945, Fig. 5A, B).

Notably, cases in our diagnostic cohort (Fig. 1B) initially erroneously discriminated by CA-125 or 4-miRNA panel were significantly reduced after reclassification by our prediction model (Fig. 5C, D). The positive predictive value (PPV = 0.926) and negative predictive value (NPV = 0.871) of this model were significantly increased compared to CA-125 (PPV = 0.706; NPV = 0.712) (Table 1). Decision curve analysis (DCA) indicated that the decision utility of 4-miRNA combined CA-125 for defining high-risk groups was superior to CA-125 or 4-miRNA alone (Fig. 5E). The quantitative evaluation of net reclassification improvement (NRI) and integrated discrimination improvement (IDI) further showed that the additional predictive power of our combined model for the residual disease was significantly improved compared with CA-125 or 4-miRNA alone (model vs CA-125, NRI = 0.471, P < 0.001; IDI = 0.538, P < 0.001; model vs 4-miRNA panel, NRI = 0.122, P = 0.001; IDI = 0.185, P = 0.003) (Table 2). Pairwise comparison of ROC curves showed that the increased AUC value of the combined model had statistical significance compared with CA-125 (AUC: 0.914, 0.679; P < 0.0001) or 4-miRNA (AUC: 0.914, 0.853; P = 0.0007) (Table 2).

**Table 1:** Performance of CA-125, 4 miRNAs, 4-miRNA panel, and 4-miRNA combined with CA-125 for predicting residual disease

Biomarker	Performance	Discovery set (n = 30)	Validation set (n = 154)
miR-320a-3p	Sensitivity	100	81.6
	Specificity	53.3	62.7
	PPV	65.2	80.0
	NPV	100	72.4
	AUC	75.6	77.2
miR-378a-3p	Sensitivity	66.7	87.4
	Specificity	80.0	50.7
	PPV	76.9	69.7
	NPV	70.6	75.6
	AUC	74.7	75.1
miR-1307-3p	Sensitivity	66.7	57.5
	Specificity	86.7	77.6
	PPV	76.9	76.1
	NPV	70.6	58.6
	AUC	77.6	68.2
let-7d-3p	Sensitivity	73.3	78.2
	Specificity	66.7	65.7
	PPV	68.8	74.2
	NPV	71.4	67.7
	AUC	69.3	78.4
4-miRNA panel	Sensitivity	93.3	83.9
	Specificity	93.3	85.1
	PPV	87.5	88.0
	NPV	92.9	80.3
	AUC	96.9	84.5
CA-125	Sensitivity	93.3	82.8
	Specificity	40.0	56.7
	PPV	50.0	70.6
	NPV	83.3	71.2
	AUC	66.2	72.3
CA-125 + 4-miRNA panel	Sensitivity	93.3	89.7
	Specificity	100.0	91.0
	PPV	100.0	92.6
	NPV	93.8	87.1
	AUC	97.3	90.3

The sensitivity, specificity, *PPV* positive predictive value, NPV negative predictive value and area under the curve (AUC) are shown as % (simple counts)

**Table 2:** Reclassification of the prediction model (4-miRNA combined CA-125) compared to CA-125 or 4-miRNA panel alone in subjects (n = 184)

Model	CA-125	CA-125 + 4-miRNA panel	P value	4-miRNA panel	4-miRNA panel + CA-125	P value
AUC	0.679	0.914	** < 0.0001**	0.853	0.914	**0.0007**
NRI	0.471		** < 0.001**	0.122		**0.001**
IDI	0.538		** < 0.001**	0.185		**0.003**

Significant P values in bold; *IDI* Integrated Discrimination Improvement, *NRI* net reclassification improvement

To further assess the model developed herein, we investigated the influence of patient backgrounds. Through postsurgical pathological reports, we found that non-R0 and R0 AOC cases were differed for stage, lymph node metastasis, and serum CA-125 levels, whereas age and P53 mutation did not differ significantly (Table 3, Additional file 1: Table S4–S6). Additionally, multivariate logistic regression analysis revealed that our newly developed 4-miRNA panel emerged as an independent risk feature for residual disease in both clinical cohorts (discovery set: OR = 2.415; 95% CI 1.779 ~ 3.279; P < 0.0001, validation set: OR = 1.866; 95% CI 1.649 ~ 2.110; P < 0.0001, Table 3). The post hoc power of our model was above 0.9. Circulating 4-miRNA expression was also significantly different between the OC and normal groups according to analysis of public datasets (GSE113486 and GSE94533) (Fig. 5F). These results indicated that the combining model of 4-miRNA and CA-125 represented a promising classifier for residual disease detection.

**Table 3:** Univariate and multivariate logistic regression analysis for residual disease in the discovery and validation cohorts

	Discovery set ( n = 30)			Validation set ( n = 154)		
	OR	95% CI	P value	OR	95% CI	P value
Univariate logistic regression analysis						
Age (years) (≥ 60 vs. < 60)	0.375	0.073 ~ 1.920	0.229	0.795	0.390 ~ 1.622	0.529
Stage (IV vs. III)	5.091	0.496 ~ 52.287	0.130	10.601	3.537 ~ 31.775	** < 0.0001**
Lymph node metastasis (positive vs. negative)	1.375	0.286 ~ 6.603	0.690	2.561	1.275 ~ 5.144	**0.0075**
P53 mutation (Positive vs. Negative)	0.762	0.179 ~ 3.241	0.712	1.167	0.519 ~ 2.623	0.710
Serum CA-125 (≥ 600 vs. < 600 U/ml)	5.688	0.939 ~ 34.458	**0.042**	4.767	2.261 ~ 10.054	** < 0.0001**
4-miRNA panel (High vs. Low risk)	91	7.348 ~ 1126.947	** < 0.0001**	29.721	12.299 ~ 71.826	** < 0.0001**
Multivariate logistic regression analysis						
Age (years) (≥ 60 vs. < 60)	1.156	0.862 ~ 1.551	0.342	0.963	0.844 ~ 1.100	0.581
Stage (IV vs. III)	0.863	0.597 ~ 1.247	0.441	1.169	1.005 ~ 1.359	**0.045**
Lymph node metastasis (Positive vs. Negative)	1.048	0.787 ~ 1.395	0.753	1.142	1.005 ~ 1.297	**0.044**
P53 mutation (Positive vs. Negative)	0.952	0.740 ~ 1.226	0.709	1.030	0.899 ~ 1.180	0.672
Serum CA-125 (≥ 600 vs. < 600 U/ml)	1.047	0.785 ~ 1.395	0.759	1.249	1.096 ~ 1.424	**0.001**
4-miRNA panel (High vs. low risk)	2.415	1.779 ~ 3.279	** < 0.0001**	1.866	1.649 ~ 2.110	** < 0.0001**

Significant P values in bold, *OR* odds ratio, *CI* confidence interval

### Tumor cell-derived sEV miRNAs contribute to the plasma sEV miRNAs signature in AOC patients

**Fig. 6:**
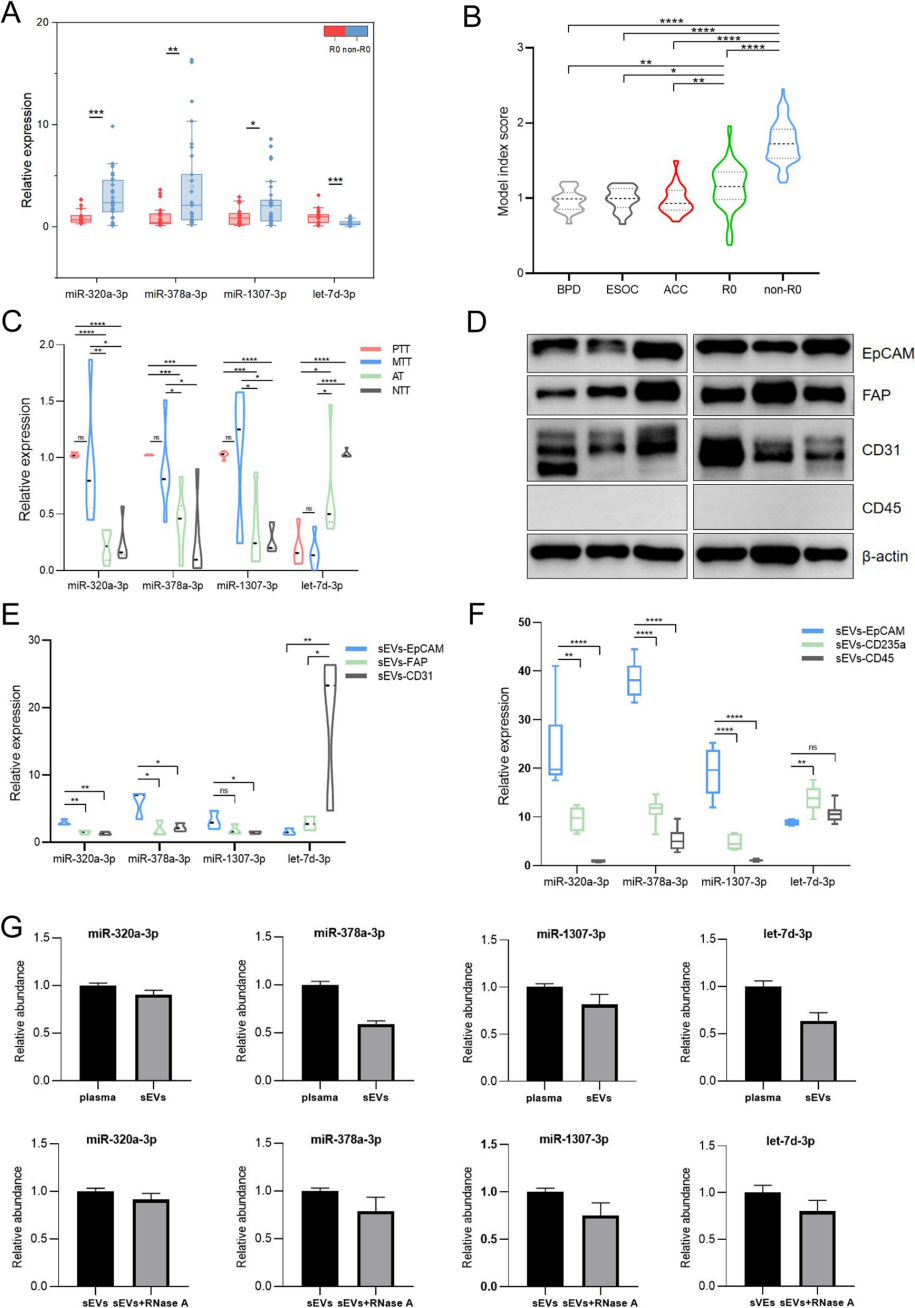
Tumor cells-derived sEVs miRNAs contribute to plasma sEVs miRNAs signature in AOC patients **A**: The 4-miRNA expressions in tumor tissue between two groups were verified by Taqman qRT-PCR (R0, n = 20; non-R0, n = 30). **B**: The 4-miRNA expressions in sEVs derived from primary tumor tissue (PTT, n = 6), metastatic tumor tissue (MTT, n = 6), adjacent tissue (AT, n = 6), or non-tumor tissue (NTT, n = 6) via Taqman qRT-PCR. **C**: Representative western blots of cell-type-specific protein in primary tumor tissue sEVs (n = 6); **D**: Taqman qRT-PCR analysis of 4-miRNA in different cell-source sEVs separated from primary tumor tissue (n = 3); **E**: Taqman qRT-PCR analysis of 4-miRNA in plasma different cell-source sEVs captured by magnetic beads sorting system (n = 6); EpCAM was chosen as epithelial ovarian cancer cells marker; FAP as cancer-associated fibroblasts marker; CD31 as endothelial cells marker; CD45 as tumor-infiltrating immune cells and leukocytes marker; CD235a as erythrocytes marker. **F:** The comparison of model index score between R0 (n = 82) or non-R0 (n = 102) patients and benign pelvic diseases (BPD, n = 21), early-stage ovarian cancer with FIGO I or II (ESOC, n = 20), and advanced colorectal cancer patients (ACC, n = 22). **G**: The relative expression levels of miR-320a-3p, miR-378a-3p, miR-1307-3p, let-7d-3p were detected from total plasma, plasma sEVs, and plasma sEVs pretreating with RNase A. miRNA, microRNA; R0, advanced ovarian cancer with no residual disease; non-R0, advanced ovarian cancer with any residual disease; sEVs, small extracellular vesicles; AOC, advanced ovarian cancer; *P < 0.05; ** < 0.01; *** < 0.001; **** < 0.0001 (unpaired t-test)

Interestingly, the expression trend of 4-miRNA in tumor tissues of AOC patients was consistent with that in circulating sEVs (Fig. 6A). In addition, for testing the differential diagnostic ability of this model, we calculated the model index score of patients with benign pelvic diseases (BPD), early-stage ovarian cancer with FIGO I or II (ESOC), and advanced colorectal cancer (ACC). Surprisingly, the model index scores of the above patients were significantly lower than AOC patients (Fig. 6B). Thus, we further investigated 4-miRNA expressions in different tissue-derived sEVs. The isolated tissue vesicles were sEVs based on characterization analysis (Additional file 1: Figures S3 and S4). The expression levels of 3 up-regulated miRNAs (miR-320a-3p, miR-378a-3p, miR-1307-3p) in primary tumor tissue (PTT) and metastatic tumor tissue (MTT)-derived tissue-sEVs were significantly higher than those in adjacent tissue (AT) and non-tumor tissue (NTT) groups, while let-7d-3p expression presented a reverse trend (Fig. 6C). There was no significant difference between PTT and MTT groups. These results demonstrated that sEVs 4-miRNA expressions were mainly affected by tumor tissue or tumor microenvironment.

We then detected the enrichment of known celltype-specific markers in the tumor tissue-sEV pool by western blot. EpCAM (epithelial cell adhesion molecule) [33], a transmembrane protein often overexpressed in epithelial carcinomas, was chosen as the epithelial ovarian cancer cells (EOCC) marker; FAP (fibroblast activation protein) [34] as the cancer-associated fibroblasts (CAFs) marker; CD31 [35] as the endothelial cells (ECs) marker and CD45 [36] as the tumor-infiltrating immune cells (TICs) marker. We observed that EpCAM, FAP, and CD31 were expressed abundantly in tissue-derived sEV fractions, whereas CD45 was almost undetectable (Fig. 6D). The results showed that the tumor tissue-sEVs pool was a relatively mixed environment, where EOCCs, CAFs, and ECs-derived sEVs were the main contributors, whereas TICs-derived sEVs were not enriched. We further sorted tissue-derived EVs from EOCCs, CAFs, and ECs by an immunoaffinity magnetic bead sorting system (MACS). Surprisingly, the expression levels of the 3 upregulated miRNAs in the EOCCs group were higher than those in the CAFs, and ECs groups, whereas let-7d-3p expression in the EOCCs group was the lowest (Fig. 6E).

Meanwhile, we were more interested in understanding the source of sEV 4-miRNA directly from circulation and excluding the effects of circulating confounders. EpCAM has long been utilized to detect circulating epithelial tumor cells [37] and their derived sEVs [13]. Previous studies showed that most circulating sEVs concentrated by ultracentrifugation in platelet-depleted plasma originated from erythrocytes and leukocytes rather than platelets or megakaryocytes as commonly thought [38]. CD235a and CD45 were assessed as erythrocyte and leukocyte markers, respectively. We captured plasma sEVs by MACS with these specific proteins. 3 up-regulated miRNAs were packaged at high concentrations in EOCCs-derived plasma EpCAM + -sEVs, while these were not detected in erythrocytes and leukocyte-derived sEV samples (Fig. 6F). Let-7d-3p expression was the lowest in the EOCCs group (Fig. 6F). Collectively, the differential expression of plasma sEV-derived 4-miRNA between the R0 and non-R0 groups was mainly dominated by tumor cells.

The Agilent 2100 Bioanalyzer results showed that the RNA content derived from plasma sEVs was decreased by about 55% compared with RNA extracted directly from total plasma, whereas RNA derived from plasma sEVs treated with RNase A was slightly decreased by about 13% (Additional file 1: Figure S5). Moreover, the RNase degradation assay showed that circulating miR-320a-3p and miR-1307-3p mainly existed in a sEVs manner, while a small part of miR-378a-3p and let-7d-3p was a free-form, and 4-miRNA in sEVs was largely not degraded after treatment with RNase A (Fig. 6G). These results revealed that circulating sEV 4-miRNA was sufficient stable and excluded possible contamination with non-sEV miRNAs during the extraction procedure.

### Target gene prediction and pathway enrichment analysis of the multi-miRNA panel

**Fig. 7:**
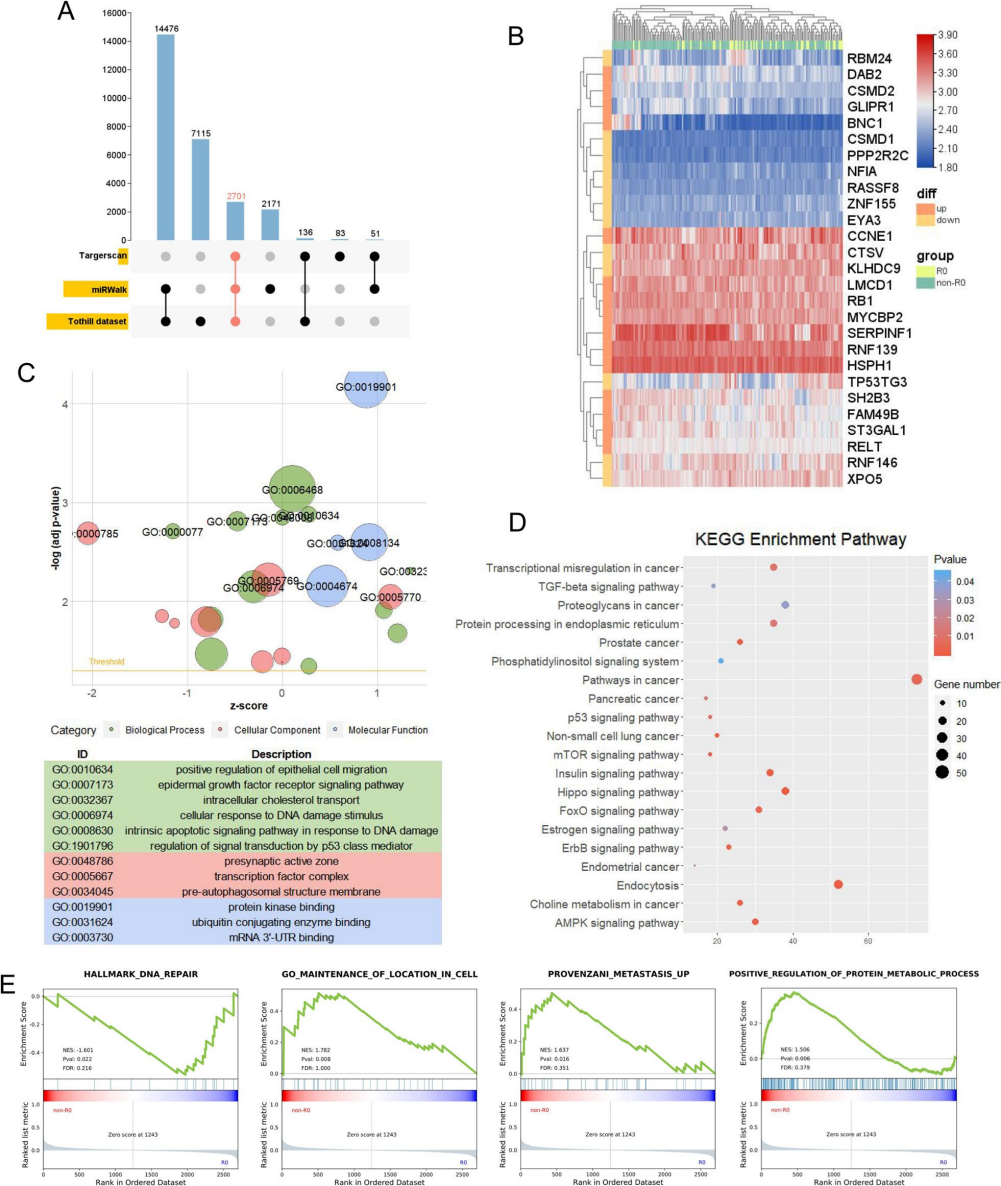
Target genes prediction and pathway enrichment analysis of the multi-miRNA panel **A**: Upset plot of 4-miRNA’s target genes via Targetscan, miRWalk, and Tothill dataset. **B**: Expression heatmap of representative 4-miRNA related target genes between R0 (n = 38) and non-R0 (n = 127) groups in the Tothill dataset. Orange represented up-regulated target genes, and yellow represented down-regulated target genes. **C**: GO enrichment analysis of target genes was performed and visualized by GOplot. Log(FC) of selected genes was taken from Tothill dataset. Z-score indicated if the biological process (biological process/cellular components/molecular function) was more likely to be increased (Z-score > 0) or decreased (Z-score < 0). The area of the circles was proportional to the number of genes in the pathway. A threshold was set as log(adj P value) > 2. **D**: A bubble plot of enriched KEGG pathway. **E**: GSEA was performed by the expression of 4-miRNA target genes between R0 and non-R0 groups in Tothill dataset. *miRNA* microRNA, *R0* advanced ovarian cancer with no residual disease; *non-R0* advanced ovarian cancer with any residual disease; *KEGG* Kyoto Encyclopedia of genes and Genomes

Furthermore, we found 2701 target genes of 4 miRNAs in common between the TargetScan, miWalk, and residual disease-associated tissue datasets (Tothill dataset, GSE61568) (Fig. 7A). 4-miRNA target genes (*TP53TG3, RB1, CCNE1, CSMD1*) closely related to HGSOC were significantly differentially expressed between the R0 and non-R0 groups in the Tothill dataset (Fig. 7B). Gene Ontology (GO) analysis showed that these mRNAs were mainly concentrated in the pathways of cell migration and DNA damage repair (Fig. 7C). In addition, we found that the *TP53*, *mTOR, FoxO,* and *ErbB*-related signaling pathways were strongly enriched in the Kyoto Encyclopedia of Genes and Genomes (KEGG) pathway categories (Fig. 7D). Gene set enrichment analysis (GSEA) also revealed that these mRNAs were significantly enriched in pathways of cell colonization and adhesion, tumor metastasis, and positive regulation of the protein metabolic process (Fig. 7E). Different but related tumor pathways of miRNA targeting suggested that 4-miRNA expression was closely associated with HGSOC progression from local to metastasis.

## Discussion

No residual disease after debulking surgery is the most critical independent prognostic factor for AOC. There is an unmet clinical need for selecting primary or interval debulking surgery using existing prediction models. In this study, we discover a distinct sEVs miRNAs profile between R0 and non-R0 AOC patient. And our aim is to find circulating sEVs miRNAs signature with the most stable expressed commonality among different AOC patients to exclude the influence of individual differences. Therefore, we have enrolled 360 patients from four clinical centers in this study. Based on it, we develop and validate a reliable and stable model of circulating sEVs miRNA panel combined with CA-125 for predicting residual disease risk in AOC patients for the first time. And this combined model can also discriminate diseases easily confounded with AOC (e. g., advanced colorectal cancer). On the other hand, Taqman qRT-PCR detection of the prediction panel is a non-invasive, safe, and easy to perform method, which is helpful for clinical application. Our prediction model can be part of a clinical standard monitoring strategy for screening high-risk AOC patients who are allowed for neoadjuvant chemotherapy instead of PDS.

NACT-IDS is the preferred alternative strategy for 30% of AOC patients with a higher disease burden who have entirely no chance for R0 resection [39]. The platinum-based chemotherapy response rate for HGSOC is as high as 80% [40]. Taylor et al. discovered that circulating tumor exosomal miRNA signatures paralleled and exhibited a strong correlation with tumor tissue-derived miRNA profiles (ranging from 0.71 to 0.90) [13]. Takahiro et al. performed the first large-scale comprehensive analysis of circulating miRNAs for the early detection of OC [14]. Their data showed that miRNA levels in early-stage OC were not significantly different from those of borderline and benign tumors, whereas miRNA levels changed dramatically in AOC. Their work revealed that circulating miRNAs could more clearly reflect AOC progression. In terms of exploring residual disease-related biomarkers, Anil K. Sood [41] found that FABP4 and ADH1B were highly expressed in the tumor tissue of non-R0 patients. Although this attempt used objective biomarkers, its limitations included not only the difficulty of obtaining tumor tissue before the operation but also the difference in gene expression rate between metastatic and primary tumor sites. There is currently no consensus on the choice of a specific circulating sEVs-derived miRNA signature to non-invasively predict residual disease in AOC.

Therefore, we performed a systemic and comprehensive analysis of the circulating sEVs miRNAs landscape in R0 and non-R0 subjects. Circulating miRNAs previously identified as diagnostic biomarkers for OC (miR-92, miR-21, miR-221, miR-200c, miR-182) were also highly expressed in our plasma sEVs samples, underlining the validity of our analysis [42]. To maximize the success rate of verification, we narrowed the scope of candidate miRNAs by the following characteristics: (1) high expression abundance; (2) conserved; and (3) targeting HGSOC closely related drive genes. Moreover, patients recruited from four clinical centers enhanced the model’s generalization.

FIGO stage and tumor burden were positively correlated with the residual disease risk of AOC patients [43]. In addition, oncogenic perturbations of tumor cells are involved in the alteration of specific miRNA species’ content in sEVs, which possibly drives cancer progression and generate novel classes of clinical biomarkers [44]. Our data showed that EOCCs-derived sEVs captured on EpCAM antibody-coated magnetic beads were the major vehicles affecting circulating 4-miRNA expressions. The model index score was positively correlated with the tumor burden of AOC patients. Although we used a novel and improved experimental method, some sEVs were still lost in the sorting process due to existing technical difficulties. Furthermore, according to the expression of miR-1307-3p and let-7d-3p in Fig. 6E, CAFs might also have some influence on panel-miRNA expression. We could not completely preclude that other cells in the tumor microenvironment or other tissues might express circulating sEVs’ 4-miRNA. During the processing of plasma samples, anticoagulants, storage temperature, and centrifugation time were strictly controlled to avoid the effects of microvesicles derived from platelet, and hemolyzed samples were removed.

A series of studies reported that circulating miR-320a could also be a potential diagnostic factor in OC [45], prostate cancer [46], and hepatocellular carcinoma [47]. Circulating miR-1307-3p is considered as a diagnostic biomarker of OC [48], breast cancer [49], and gastric cancer [50]. Circulating let-7d often emerged as a diagnostic molecular that is down-regulated in pancreatic cancer [51], hepatocellular carcinoma [52], and cervical cancer [53]. Meanwhile, there have been relatively fewer studies on miR-378a-3p as a cancer biomarker. To pin down the mechanisms and pathogenic relevance of 4-miRNA, further studies will be needed in the future.

CA-125 is dramatically elevated in BPDs such as tuberculous peritonitis and theca follicular fibroma [54]. In addition, ACC with extensive pelvis metastasis is also prone to confusion with AOC; colonoscopy is normally needed to differentiate the two. However, our model could distinguish AOC from these diseases non-invasively (Fig. 6B). Furthermore, a key prerequisite for the molecular signature with a potential clinical application is a fast, robust, and easy-to-perform laboratory assay. Our TaqMan qRT-PCR validation of the circulating sEVs miRNA panel is a vital step in this direction [11, 55]. This testing panel, as an affordable approach, is expected to provide benefits for AOC patients in developing countries.

## Limitations

We will further perform additional survival analysis when the needed terminal events have been reached. Additionally, we collected plasma samples from patients who received NACT after every round of chemotherapy to detect changes in the model index score to further explore the best time for IDS. Meanwhile, we may employ droplet digital PCR in the subsequent clinical transformation.

## Conclusions

Overall, for the first time, we established a reliable and objective model composed of circulating tumor-derived sEVs 4-miRNA and CA-125 for predicting residual disease risk in AOC patients, which could accurately assess the triage of PDS versus NACT-IDS. This model, as a tool for non-invasive liquid biopsy, has great potential to be part of clinical monitoring strategies for screening high-risk patients with residual disease.

### Supplementary Information

**Additional file 1: Table S1.** 51 selected miRNAs ranked by logFC × P value (miRNA-seq data). **Table S2.** Fold changes, corresponding P, and AUC values of 15 candidate miRNAs in the discovery cohort. **Table S3.** PCR primer and probe sequences. **Table S4.** Baseline characteristics of the patients in screening cohort. **Table S5.** Baseline characteristics of the patients in discovery cohort. **Table S6.** Baseline characteristics of the patients in validation cohort. **Figure S1.** The flowdiagram of the analysis through the three cohort. **Figure S2.** Identification of sEVs extracted from AOC patients’ plasma (western blotting). **Figure S3.** Identification of sEVs extracted from AOC patients’ tissue (TEM/NTA). **Figure S4.** Identification of sEVs extracted from AOC patients’ tissue (western blotting). **Figure S5.** Plasma content characteristic verification of 4-miRNA.

## Data Availability

RNA sequence data of all samples were publically stored in the Gene Expression Omnibus of NCBI, GSE223126 (https://www.ncbi.nlm.nih.gov/geo/query/acc.cgi?acc=GSE223126). The accession numbers of previously published datasets involved in our study were GSE113486 [26], GSE94533 [27], and GSE61568 [22].

## Abbreviations

R0 resection: No: residual disease
AOC: Advanced ovarian cancer
sEVs: Small extracellular vesicles
miRNA: MicroRNAs
LASSO: Least absolute shrinkage and selection operator
NRI: Net reclassification improvement
IDI: Integrated discrimination improvement
PDS: Primary debulking surgery
TEM: Transmission electron microscopy
DEMs: Differentially expressed miRNAs
R0: Advanced ovarian cancer with no residual disease
non-R0: Advanced ovarian cancer with any residual disease
AUC: Area under the receiver operating characteristic curve
ROC: Receiver operating characteristic curve
KEGG: Kyoto Encyclopedia of genes and Genomes
