# Supplementary material for: Circulating small extracellular vesicles microRNAs plus CA-125 for treatment stratification in advanced ovarian cancer
Source: J Transl Med. 2023 Dec 22;21:927. doi: 10.1186/s12967-023-04774-4 (PMC10740240; doi:10.1186/s12967-023-04774-4)
Supplement: Supplementary file 1 — Additional file 1: Table S1. 51 selected miRNAs ranked by logFC × P value (miRNA-seq data). Table S2. Fold changes, corresponding P, and AUC values of 15 candidate miRNAs in the discovery cohort. Table S3. PCR primer and probe sequences. Table S4. Baseline characteristics of the patients in screening cohort. Table S5. Baseline characteristics of the patients in discovery cohort. Table S6. Baseline characteristics of the patients in validation cohort. Figure S1. The flowdiagram of the analysis through the three cohort. Figure S2. Identification of sEVs extracted from AOC patients’ plasma (western blotting). Figure S3. Identification of sEVs extracted from AOC patients’ tissue (TEM/NTA). Figure S4. Identification of sEVs extracted from AOC patients’ tissue (western blotting). Figure S5. Plasma content characteristic verification of 4-miRNA. [file 12967_2023_4774_MOESM1_ESM.pdf]

Supplementary Tables

Table S1. 51 selected miRNAs ranked by logFC × P value (miRNA-seq data)

miRNA	logFC	P value
hsa-miR-1268b	6.524567708	0.000000533
hsa-miR-1268a	6.525002054	0.000000699
hsa-miR-4466	5.96884733	0.00000228
hsa-miR-320e	4.444697106	0.00000244
hsa-miR-320a-3p	3.996376421	0.00000607
hsa-miR-3180-3p	5.201573618	0.00000821
hsa-miR-3180	5.201573618	0.00000821
hsa-miR-320b	4.144389384	0.0000107
hsa-miR-378c	3.746973292	0.0000133
hsa-miR-4429	4.140066797	0.0000166
hsa-miR-204-3p	4.444515351	0.0000205
hsa-miR-1228-5p	5.155282818	0.0000275
hsa-miR-378d	3.500830991	0.0000371
hsa-miR-210-3p	3.751611987	0.0000507
hsa-miR-320c	3.913906983	0.0000647
hsa-miR-320d	3.41102307	0.0000977
hsa-miR-483-5p	5.51086822	0.000159712
hsa-miR-193a-5p	3.054098377	0.000190597
hsa-miR-378f	3.019575676	0.00028609
hsa-miR-193b-5p	3.181715872	0.000356465
hsa-miR-92b-5p	3.168144484	0.00046643
hsa-miR-23a-5p	3.985625677	0.000515949
hsa-miR-4508	3.161609242	0.000538284
hsa-miR-296-3p	4.583428706	0.000553897
hsa-miR-378a-3p	2.441412659	0.000781566
hsa-miR-574-5p	2.867134178	0.00194959
hsa-miR-192-5p	2.356751319	0.004978032
hsa-miR-200c-3p	2.493437702	0.006004055
hsa-miR-423-5p	2.234052743	0.006706894
hsa-miR-708-5p	-4.839038271	0.00740506

miRNA, microRNA; FC, fold change.

Table S1. 51 selected miRNAs ranked by logFC × P value (miRNA-seq data)

miRNA	logFC	P value
hsa-miR-576-3p	2.267916683	0.007767367
hsa-miR-27a-5p	-2.079362343	0.008138721
hsa-miR-145-3p	-1.983973431	0.009036106
hsa-miR-378i	1.786939288	0.010810812
hsa-miR-1307-3p	1.876802327	0.014652655
hsa-miR-134-5p	1.965724978	0.014909171
hsa-miR-27a-3p	-1.73414236	0.016289525
hsa-let-7d-3p	-1.830102743	0.016358829
hsa-miR-200b-3p	2.08701507	0.017105133
hsa-miR-425-3p	2.182146297	0.021104559
hsa-miR-1908-5p	1.914822438	0.027228563
hsa-miR-148a-5p	1.763492298	0.027638532
hsa-miR-133a-3p	2.343411857	0.031330922
hsa-miR-629-5p	1.889319093	0.03193781
hsa-miR-671-5p	2.265121056	0.032108301
hsa-miR-4488	1.914908497	0.033640396
hsa-miR-223-5p	-1.662332383	0.034344872
hsa-miR-10b-5p	-1.579421923	0.043898787
hsa-miR-338-5p	-1.494317432	0.04428883
hsa-miR-3960	2.40192849	0.044384245
hsa-miR-451a	-1.329068528	0.049320672

miRNA, microRNA; FC, fold change.

Table S2. Fold changes, corresponding P, and AUC values of 15 candidate miRNAs in the discovery cohort

miRNA	FC	P value	AUC
miR-1307-3p	3.315	**0.009**	0.776
miR-320a-3p	2.186	**0.0199**	0.756
miR-378a-3p	2.943	**0.0217**	0.747
miR-27a-3p	3.464	**0.0274**	0.636
miR-320b	2.094	**0.0387**	0.733
miR-200c-3p	9.843	**0.0391**	0.711
let-7d-3p	0.368	**0.0421**	0.693
miR-423-5p	2.126	**0.0427**	0.704
miR-451a	3.114	0.0512	0.693
miR-193a-5p	1.781	0.0583	0.667
miR-223-5p	2.77	0.0671	0.684
miR-192-5p	4.927	0.0743	0.733
miR-483-5p	2.533	0.1063	0.647
miR-210-3p	1.613	0.1234	0.664
miR-204-3p	0.988	0.9719	0.578

Significant P values in bold. miRNA, microRNA; FC, fold change; AUC, area under the receiver operating characteristic curve.

Table S3. PCR primer and probe sequences

miRNA	Sequences
hsa-miR-320a-3p-RT	GTCGTATCCAGTGCAGGGTCCGAGGTATTCGCACTGGATACGACTCGCCC
hsa-miR-320a-3p-F	ACGCAAAAGCTGGGTTGAGA
hsa-miR-320a-3p-P	TTCGCACTGGATACGACTCGCCC
hsa-miR-320b-RT	GTCGTATCCAGTGCAGGGTCCGAGGTATTCGCACTGGATACGACTTGCCC
hsa-miR-320b-F	ACGCAAAAGCTGGGTTGAGA
hsa-miR-320b-P	TTCGCACTGGATACGACTTGCCC
hsa-miR-204-3p-RT	GTCGTATCCAGTGCAGGGTCCGAGGTATTCGCACTGGATACGACACGTCC
hsa-miR-204-3p-F	AGGTGCTGGGAAGGCAAAG
hsa-miR-204-3p-P	TTCGCACTGGATACGACACGTCC
hsa-miR-210-3p-RT	GTCGTATCCAGTGCAGGGTCCGAGGTATTCGCACTGGATACGACTCAGCCGC
hsa-miR-210-3p-F	AGTCCTGTGCGTGTGACA
hsa-miR-210-3p-P	TCGCACTGGATACGACTCAGCCGC
hsa-miR-483-5p-RT	GTCGTATCCAGTGCAGGGTCCGAGGTATTCGCACTGGATACGACCTCCCT
hsa-miR-483-5p-F	ACGCAAGACGGGAGGAAAGA
hsa-miR-483-5p-P	TTCGCACTGGATACGACCTCCCT
hsa-miR-193a-5p-RT	GTCGTATCCAGTGCAGGGTCCGAGGTATTCGCACTGGATACGACTCATCTC
hsa-miR-193a-5p-F	ACTGGGTCTTTGCGGGC
hsa-miR-193a-5p-P	TTCGCACTGGATACGACTCATCTC
hsa-miR-378a-3p-RT	GTCGTATCCAGTGCAGGGTCCGAGGTATTCGCACTGGATACGACGCCTTC
hsa-miR-378a-3p-F	CCGACTGGACTTGGAGTCA
hsa-miR-378a-3p-P	TCGCACTGGATACGACGCCTTC
hsa-miR-192-5p-RT	GTCGTATCCAGTGCAGGGTCCGAGGTATTCGCACTGGATACGACGGCTGT
hsa-miR-192-5p-F	ACCGCCTGACCTATGAATTG
hsa-miR-192-5p-P	TCGCACTGGATACGACGGCTGT
hsa-miR-200c-3p-RT	GTCGTATCCAGTGCAGGGTCCGAGGTATTCGCACTGGATACGACTCCATC
hsa-miR-200c-3p-F	ACGCTAATACTGCCGGGTAAT
hsa-miR-200c-3p-P	TTCGCACTGGATACGACTCCATC
hsa-miR-423-5p-RT	GTCGTATCCAGTGCAGGGTCCGAGGTATTCGCACTGGATACGACAAAGTCTC
hsa-miR-423-5p-F	ACTGAGGGGCAGAGAGC
hsa-miR-423-5p-P	TTCGCACTGGATACGACAAAGTCTC

RT, specific RT primer; F, forward primer; R, reverse primer; UR, universal reverse primer; P, probe.

Table S3. PCR primer and probe sequences

miRNA	Sequences
hsa-miR-27a-3p-RT	GTCGTATCCAGTGCAGGGTCCGAGGTATTCGCACTGGATACGACGCGGAACT
hsa-miR-27a-3p-F	ACGCTTCACAGTGGCTAAG
hsa-miR-27a-3p-P	CACTGGATACGACGCGGAACT
hsa-miR-1307-3p-RT	GTCGTATCCAGTGCAGGGTCCGAGGTATTCGCACTGGATACGACCACGAC
hsa-miR-1307-3p-F	AACTCGGCGTGGCGTCG
hsa-miR-1307-3p-P	TTCGCACTGGATACGACCACGAC
hsa-let-7d-3p-RT	GTCGTATCCAGTGCAGGGTCCGAGGTATTCGCACTGGATACGACAGAAAGGC
hsa-let-7d-3p-F	ACGCCTATACGACCTGCT
hsa-let-7d-3p-P	TCGCACTGGATACGACAGAAAGGC
hsa-miR-223-5p-RT	GTCGTATCCAGTGCAGGGTCCGAGGTATTCGCACTGGATACGACAACTCAG
hsa-miR-223-5p-F	ACCGCCGTGTATTTGACAAG
hsa-miR-223-5p-P	TTCGCACTGGATACGACAACTCAG
hsa-miR-451a-RT	GTCGTATCCAGTGCAGGGTCCGAGGTATTCGCACTGGATACGACAACTCAG
hsa-miR-451a-F	CCGCCAAACCGTTACCATTA
hsa-miR-451a-P	TTCGCACTGGATACGACAACTCAG
cel-miR-39-RT	GTCGTATCCAGTGCAGGGTCCGAGGTATTCGCACTGGATACGACCAAGCT
cel-miR-39-F	CGCTCACCGGGTGTAAATC
cel-miR-39-P	ATTCGCACTGGATACGACCAAGCT
U6-RT	AACGCTTCACGAATTTGCGT
U6-F	AACGCTTCACGAATTTGCGT
U6-R	CTCGCTTCGGCAGCACA
U6-P	AGAAGATTAGCATGGCCCCTGCGCA
qPCR-UR	GTGCAGGGTCCGAGGT

RT, specific RT primer; F, forward primer; R, reverse primer; UR, universal reverse primer; P, probe.

Table S4. Baseline characteristics of the patients in screening cohort

Characteristics	Number of cases	R0 (n=17)	non-R0 (n=20)
**Age (years)**			
<60	25	12(70%)	13(65%)
≥60	12	5(30%)	7(35%)
**FIGO Stage**			
III	30	16(94%)	14(70%)
IV	7	1(6%)	6(30%)
**Preoperative CA-125 (U/mL)**			
<600	11	8(47%)	3(15%)
≥600	26	9(53%)	17(85%)
**Lymph node metastasis**			
Positive	24	10(59%)	14(70%)
Negative	13	7(41%)	6(30%)
**P53 mutation**			
Positive	22	11(65%)	12(60%)
Negative	14	6(35%)	8(40%)

Table S5. Baseline characteristics of the patients in discovery cohort

Characteristics	Number of cases	R0 (n=15)	non-R0 (n=15)
**Age (years)**			
<60	21	9(60%)	12(80%)
≥60	9	6(40%)	3(20%)
**FIGO Stage**			
III	26	15(100%)	11(73%)
IV	4	0(0)	4(27%)
**Preoperative CA-125 (U/mL)**			
<600	9	7(47%)	2(13%)
≥600	21	8(53%)	13(86%)
**Lymph node metastasis**			
Positive	21	10(67%)	11(73%)
Negative	9	5(33%)	4(27%)
**P53 mutation**			
Positive	17	9(60%)	8(53%)
Negative	13	6(40%)	7(47%)

Table S6. Baseline characteristics of the patients in validation cohort

Characteristics	Number of cases	R0 (n=67)	non-R0 (n=87)
**Age (years)**			
<60	112	46(69%)	66(76%)
≥60	42	21(31%)	21(24%)
**FIGO Stage**			
III	115	64(96%)	51(59%)
IV	39	3(4%)	36(41%)
**Preoperative CA-125 (U/mL)**			
<600	46	32(48%)	14(16%)
≥600	108	35(52%)	73(84%)
**Lymph node metastasis**			
Positive	97	33(49%)	64(74%)
Negative	57	34(50%)	23(26%)
**P53 mutation**			
Positive	78	37(55%)	41(47%)
Negative	76	30(45%)	46(53%)

Supplementary Figures

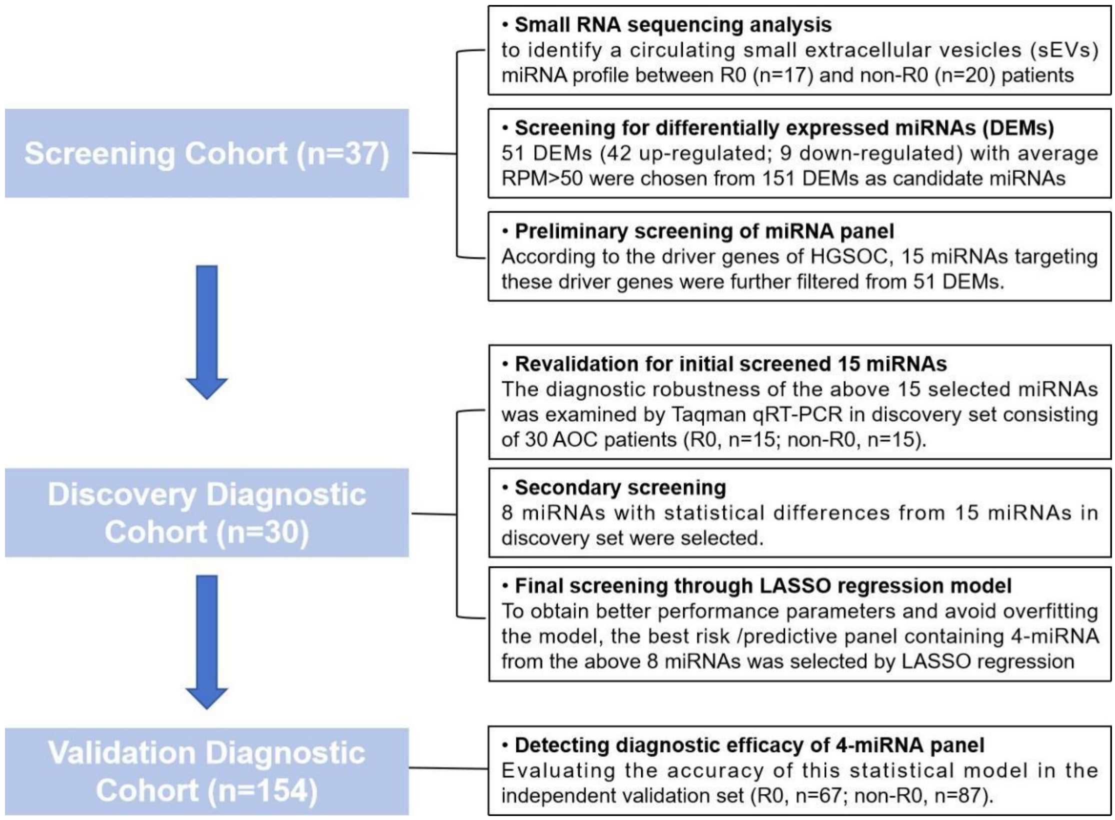

Figure S1. The flowdiagram of the analysis through the three cohort. Flowdiagram of study design was prepared to establish a diagnostic model for predicting residual disease risk in AOC patients. AOC, advanced ovarian cancer.

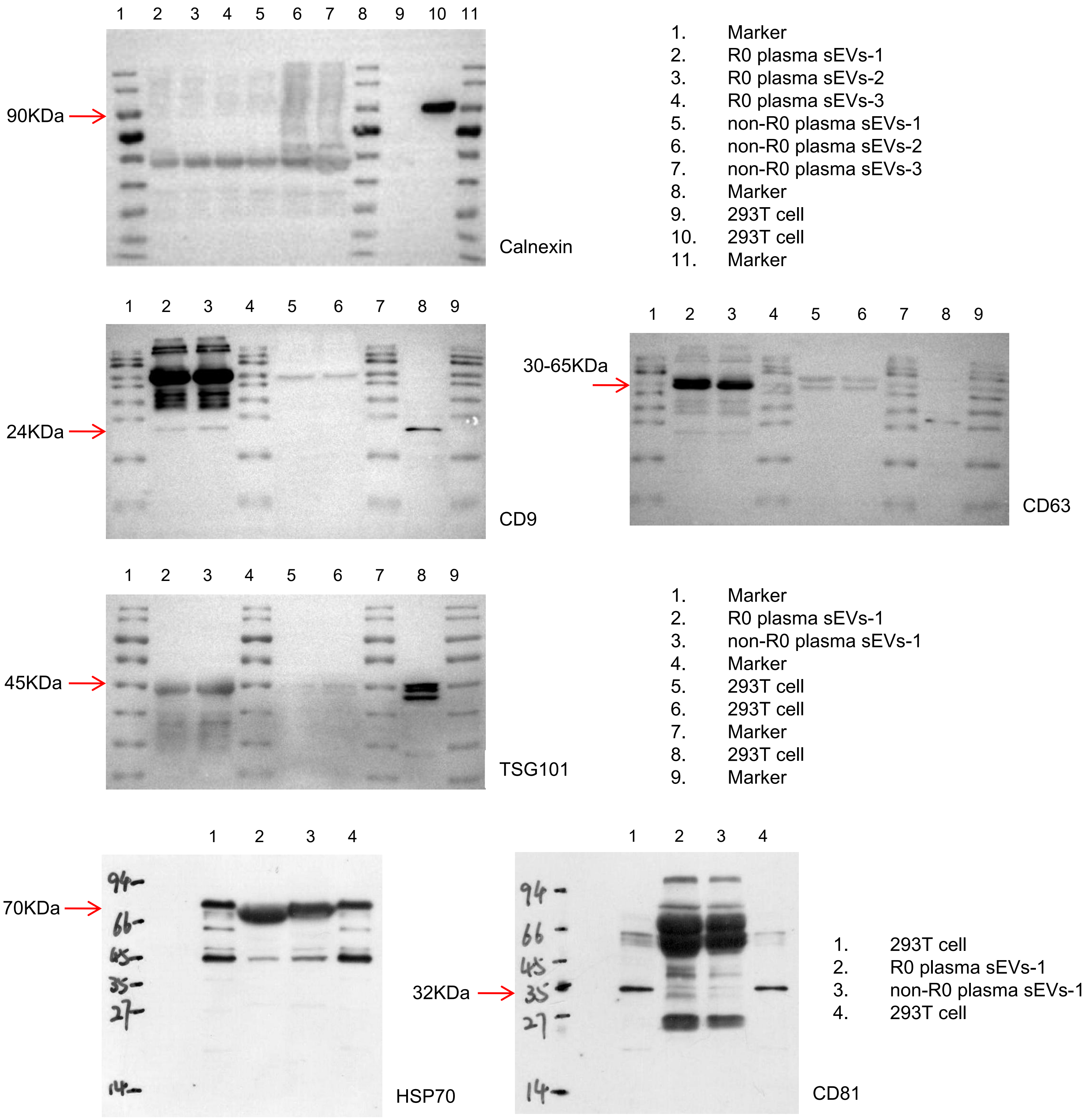

Figure S2. Identification of sEVs extracted from AOC patients' plasma (western blotting)

The sEVs typically protein markers CD9, CD63, TSG101, HSP70, CD81, and Calnexin were detected by western blotting analysis. sEVs, small extracellular vesicles; R0, advanced ovarian cancer patients with no residual disease; non-R0, advanced ovarian cancer patients with any residual disease.

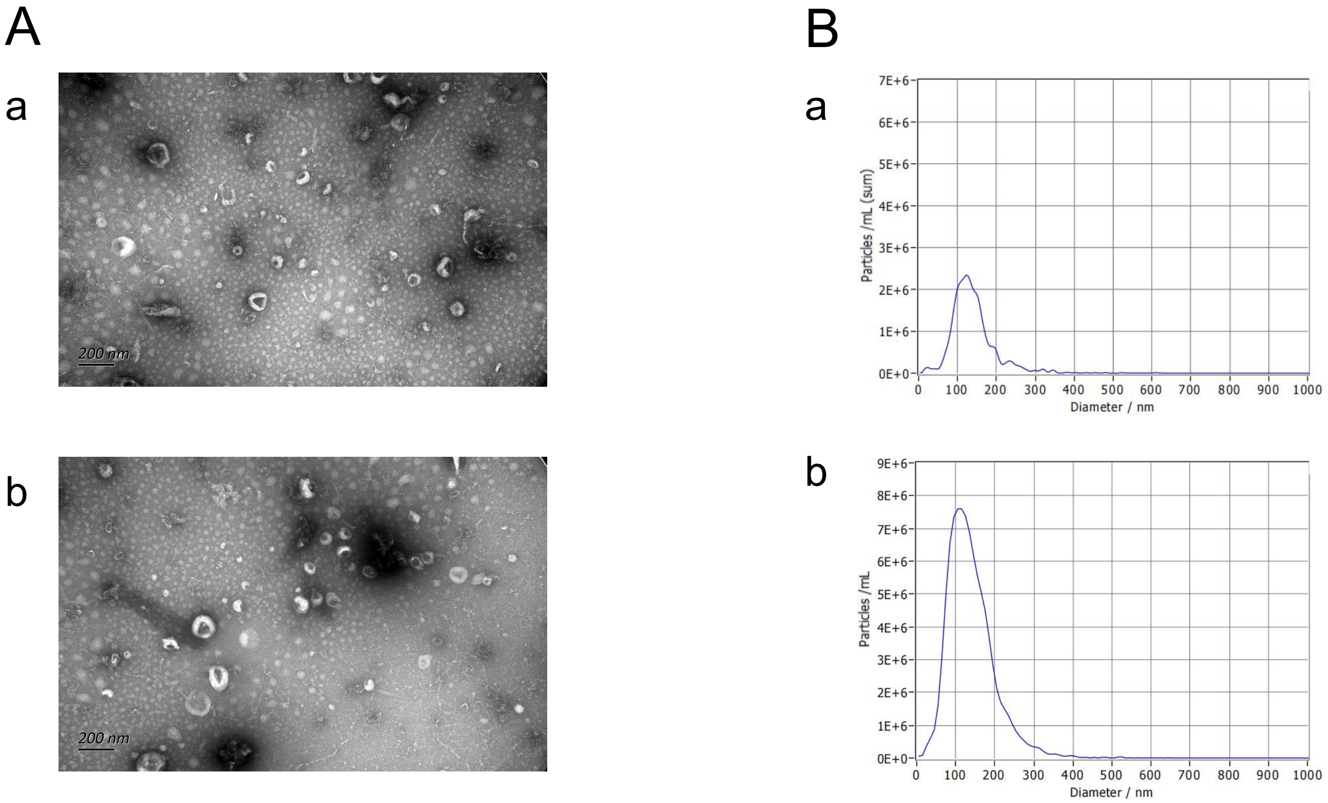

Figure S3. Identification of sEVs extracted from AOC patients' tissue (TEM/NTA)

(A) Transmission electron microscopy (TEM) images of vesicles isolated from R0 (a) and non-R0 (b) patients' tissue. Scale bar, 200 nm. (B) Nanoparticle tracking analysis (NTA) determined the size distribution of vesicles isolated from R0 (a) and non-R0 (b) patients' tissue. R0, advanced ovarian cancer patients with no residual disease; non-R0, advanced ovarian cancer patients with any residual disease.

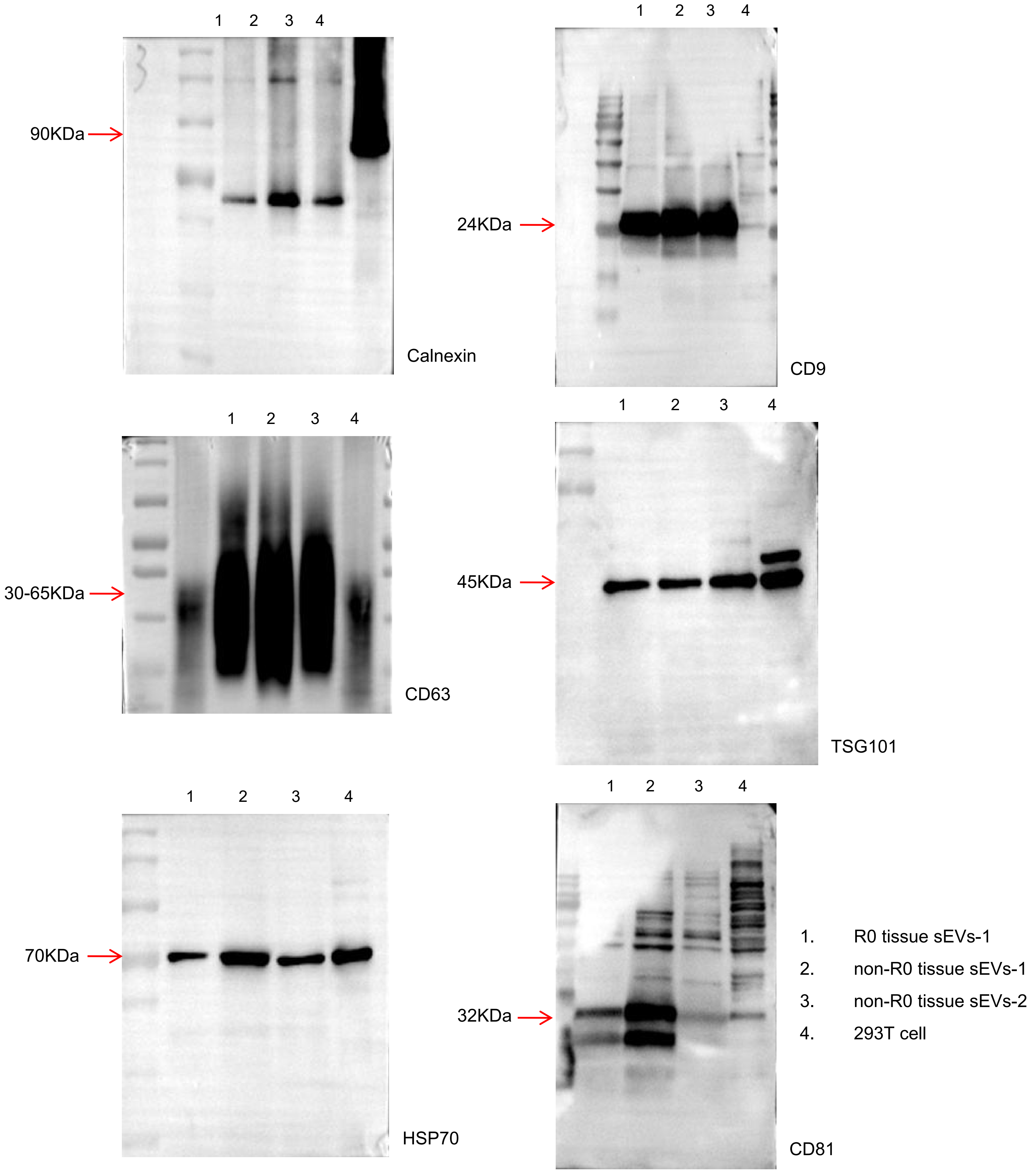

Figure S4. Identification of sEVs extracted from AOC patients' tissue (western blotting)

The sEVs typically protein markers CD9, CD63, TSG101, HSP70, CD81, and Calnexin were detected by western blotting analysis. sEVs, small extracellular vesicles; R0, advanced ovarian cancer patients with no residual disease; non-R0, advanced ovarian cancer patients with any residual disease.

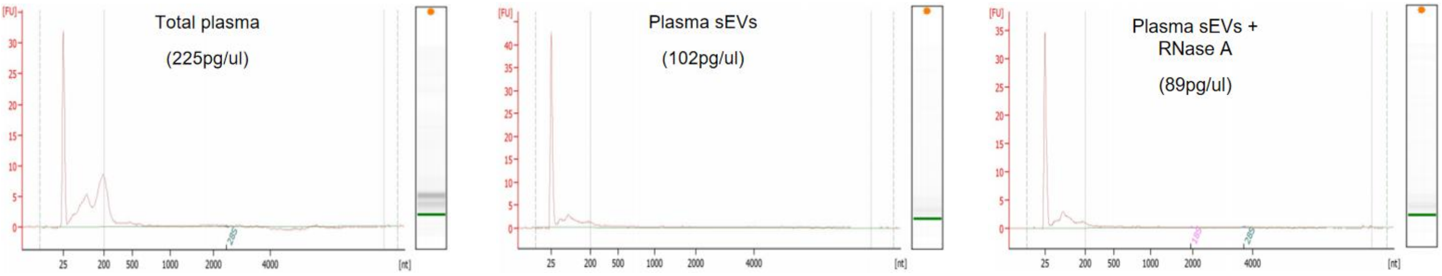

Figure S5. Plasma content characteristic verification of 4-miRNA

Representative Agilent 2100 Bioanalyzer results for RNA extracted from total plasma, plasma sEVs, and RNase A-treated plasma sEVs. miRNA, microRNA; sEVs, small extracellular vesicles.
